# Evolving incretin-based therapies in Japan: optimizing treatment strategies for diverse clinical and socioeconomical profiles in type 2 diabetes

**DOI:** 10.1007/s13340-025-00818-w

**Published:** 2025-05-15

**Authors:** Yohei Seno, Eri Ikeguchi, Daisuke Yabe

**Affiliations:** Department of Diabetes, Endocrinology and Nutrition, Kyoto University Graduate School of Medicine, 54 Shogoin-kawahara-cho, Sakyo-ku, Kyoto, 606-8507 Japan

**Keywords:** Incretins, DPP-4 inhibitors, GLP-1 receptor agonists, GIP/GLP-1 receptor agonist

## Abstract

The management of type 2 diabetes has evolved significantly with the advent of incretin-based therapies, particularly dipeptidyl peptidase-4 inhibitors and glucagon-like peptide-1 receptor agonists. In Japan, where over 70% of individuals with diabetes are aged 65 or older and often exhibit a non-obese phenotype with impaired insulin secretion, dipeptidyl peptidase-4 inhibitors remain a cornerstone therapy due to their ability to enhance insulin secretion without increasing hypoglycemia risk. Meanwhile, in younger adults with obesity, glucagon-like peptide-1 receptor agonists play a crucial role by improving glycaemia, promoting weight loss, and offering cardiovascular and renal protection. A major breakthrough in 2023 was the introduction of glucose-dependent insulinotropic polypeptide/glucagon-like peptide-1 receptor agonist tirzepatide, which activates both receptors and has shown superior glucose-lowering and weight-reducing effects in both clinical trials and real-world Japanese settings. However, incretin-based therapies are frequently associated with gastrointestinal side effects, and concerns remain regarding their potential impact on pancreatic and biliary diseases as well as frailty and sarcopenia in older adults. In addition, inappropriate discontinuation of insulin following incretin therapy initiation has led to severe outcomes, emphasizing the need for careful clinical decision-making beyond trial data. Emerging incretin-related therapies are under investigation for obesity and metabolic disorders including type 2 diabetes. While these agents hold promise for enhanced metabolic, weight, and cardiorenal benefits, their long-term safety and applicability require further study. To optimize therapeutic strategies, adherence to evidence-based guidelines, such as the "Recommendations for the Safe Use of Incretin-Related Agents, Second Edition" by the Japanese Diabetes Society, is essential.

## Introduction

**Fig. 1:**
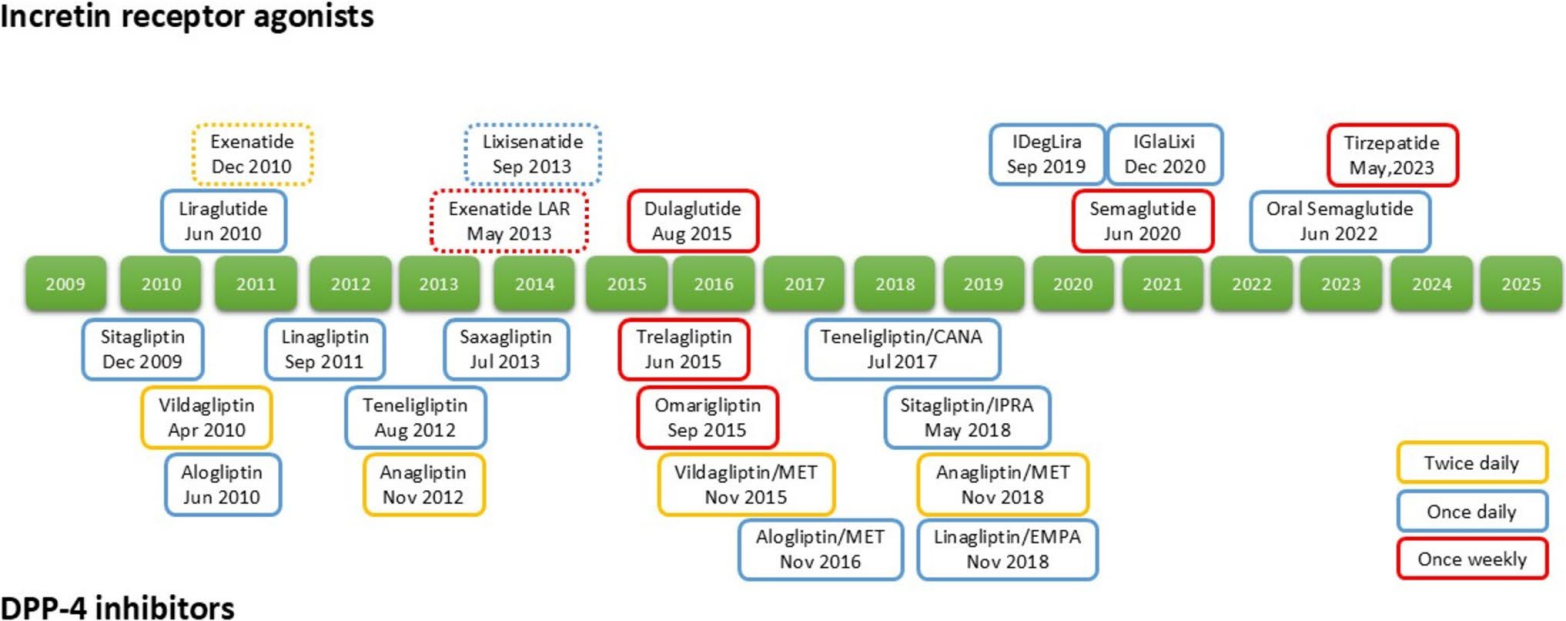
Incretin-based drugs approved in Japan. Incretin-based drugs approved in Japan are shown. The market launch year is based on the package insert of each formulation. Dashed lines indicate discontinued formulation as of March 2025. Yellow, blue and red lines indicate twice daily, once daily and once weekly formulation, respectively. CANA, canagliflozin; DPP-4, dipeptidyl peptidase-4; EMPA, empagliflozin; iDegLira, insulin degludec/liraglutide; IGla/Lixi, insulin glargine/lixisenatide; iIPRA, ipragliflozin; MET, metformin

Treatments for type 2 diabetes have evolved remarkably, allowing for the selection of therapeutic agents tailored to individual conditions, comorbidities, lifestyle, and preferences. Particularly since 2009, the introduction of dipeptidyl peptidase-4 (DPP-4) inhibitors and glucagon-like peptide-1 (GLP-1) receptor agonists (RAs) has significantly transformed diabetes care in Japan (Fig. 1) [1]. These medications have been groundbreaking as they can correct the characteristic insulin secretion deficiency observed in Japan and East Asian countries, without increasing the risk of hypoglycemia [2–4]. Furthermore, GLP-1 RAs not only improve glycaemia but also provide additional benefits such as body weight reduction and protective effects against cardiovascular disease and diabetic kidney disease (DKD), and metabolic dysfunction-associated steatotic liver disease (MASLD)[5–7]. In 2023, the advent of glucose-dependent insulinotropic polypeptide (GIP)/GLP-1 receptor agonist tripeptide, which activates both the GIP and GLP-1 receptors, marked another milestone in type 2 diabetes management in Japan (Fig. 1). Tripeptide has demonstrated significant glucose-lowering and weight-reducing effects, not only in clinical trials but also in Japanese real-world settings [8–12]. On the other hand, GLP-1 RAs and GIP/GLP-1 receptor agonist tripeptide are frequently associated with gastrointestinal side effects, such as nausea, vomiting, and diarrhea. In addition, concerns have been raised about their potential impact on biliary diseases and frailty or sarcopenia in older adults with type 2 diabetes (Table 1) [13]. Furthermore, there have been reports of individuals with insulin-dependent diabetes discontinuing insulin injections after initiating these agents, leading to fatal outcomes (Table 1) [14]. Considering these aspects, it is essential to make careful treatment decisions not solely based on clinical trial results with homogeneous populations but also by incorporating real-world clinical data. Here we overview the glucose-lowering and weight-reducing effects of incretin-based therapies, discussing the effective and safety use of DPP-4 inhibitors, GLP-1 RAs, and GIP/GLP-1 receptor agonist tripeptide in Japanese individuals with type 2 diabetes. In addition, it introduces emerging incretin-related therapies currently under development in Japan.

**Table 1:** Seven recommendations for the safe use of incretin-related agents, second edition issued by the committee on the safe use of medications, the Japan Diabetes Society.

1. Concomitant use of incretin-based drugs and hypoglycemia-prone medications
2. Transitioning from insulin to GLP-1 receptor agonists or GIP/GLP-1 receptor agonists
3. Use of GLP-1 and GIP/GLP-1 receptor agonists in older adults at risk for sarcopenia and frailty
4. Incretin-based drugs and bullous pemphigoid
5. Incretin-based drugs and pancreatic disease
6. Incretin-based drugs and biliary diseases
7. Incretin-based drugs and RS3PE syndrome

Comprehensive details for each recommendation in Japanese can be accessed at the following website: https://www.jds.or.jp/uploads/files/recommendation/incretin.pdf. GIP, glucose-dependent insulinotropic polypeptide; GLP-1, glucagon-like polypeptide-1; RS3PE, remitting seronegative symmetrical synovitis with pitting edema

## DPP-4 inhibitors

**Fig. 2:**
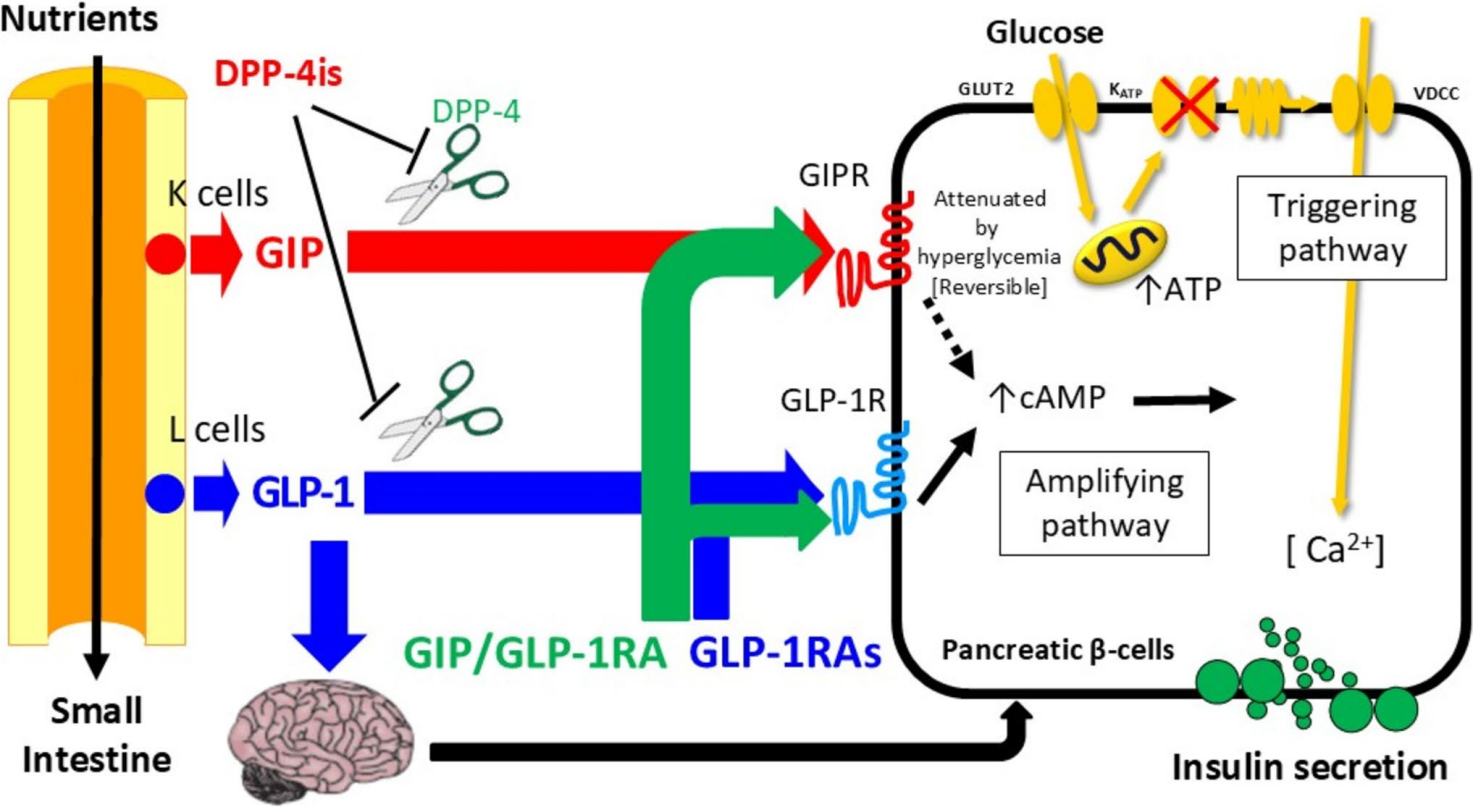
Enhancement of glucose-dependent insulin secretion by incretin-based medications. Dipeptidyl peptidase-4 inhibitors (DPP-4is) prevent the degradation of glucose-dependent insulinotropic polypeptide (GIP) and glucagon-like peptide-1 (GLP-1) by dipeptidyl peptidase-4 (DPP-4). This increases the plasma levels of biologically intact GIP and GLP-1, thereby enhancing glucose-dependent insulin secretion from pancreatic β-cells via the GIP receptor (GIPR) and GLP-1 receptor (GLP-1R). Increased biologically intact GLP-1 is also considered to enhance glucose-dependent insulin secretion via autonomic nervous system. GLP-1 receptor agonists (GLP-1RAs) and dual GIP/GLP-1 receptor agonist (GIP/GLP-1 RA) are resistant to DPP-4-mediated degradation, further amplifying glucose-dependent insulin secretion through GIPR and GLP-1R. Notably, GIPR signaling is attenuated in the presence of chronic hyperglycemia, but this impairment can be reversed with glycemic normalization

A variety of DPP-4 inhibitors are available, differing in dosing frequency, metabolism, and excretion pathways, allowing for individualized selection based on renal function, liver function, and lifestyle considerations in individuals with type 2 diabetes (Fig. 1). These agents enhance levels of biologically active GIP and GLP-1 by preventing their degradation by DPP-4, thereby stimulating glucose-dependent insulin secretion and improving glycemic control (Fig. 2) [15]. Type 2 diabetes in Japan and East Asian countries is often characterized by non-obesity and impaired insulin secretion, contrasting with the obesity-driven insulin resistance more commonly seen in Caucasian populations [2]. Consequently, both DPP-4 inhibitors and GLP-1 RAs have demonstrated greater glucose-lowering effects among East Asians [2–4]. Notably, a genetic variant of the GLP-1 receptor that enhances receptor sensitivity to GLP-1 is more prevalent in East Asians, potentially contributing to the superior glucose-lowering effects observed with these therapies [16, 17]. In addition, dietary patterns may influence treatment efficacy. Individuals consuming diets rich in saturated fats have shown attenuated glycemic responses and body weight gain following DPP-4 inhibitor initiation [18]. This may be attributed to enhanced energy storage in adipose tissue mediated by endogenous GIP, whose activity is upregulated by DPP-4 inhibition [19]. This finding may also explain the greater glucose-lowering effects of DPP-4 inhibitors in East Asian populations, where saturated fat intake is generally lower than in Western countries [20].

DPP-4 inhibitors have a low risk of hypoglycemia when used as monotherapy and can be safely prescribed to older adults with type 2 diabetes in Japan [13, 21]. However, their combination with sulfonylureas (SUs) increases the risk of severe hypoglycemia, necessitating careful dose adjustments, especially in older adults and those with impaired renal function [22]. Dose reduction of SUs is typically recommended when initiating a DPP-4 inhibitor, though immediate adjustment may not be required if the SU dose is already low. Glycemic control should be reassessed before further modifications. Studies investigating the interactions between certain SUs and EPAC2, a key mediator of incretin signaling, suggest that glimepiride and glibenclamide synergistically enhance insulin secretion when combined with DPP-4 inhibitors [23–26]. This potentiation is associated with a higher incidence of severe hypoglycemia compared to gliclazide—a trend evident in the distribution of these agents among Japanese individuals who experience severe hypoglycemia following the initiation of sitagliptin [22].

Concerns regarding an increased risk of acute pancreatitis have emerged based on global studies and post-marketing surveillance data [13, 27]. Large-scale Japanese studies including our analysis on nationwide claims database have not demonstrated a causal link between DPP-4 inhibitors and acute pancreatitis [28], possibly due to differences in genetic predisposition, dietary habits, and lower baseline pancreatic fat accumulation. The association between DPP-4 inhibitors and pancreatic cancer remains inconclusive in global studies [27], and this uncertainty extends to Japanese populations as large Japanese cohort studies have not demonstrated a statistically significant increase in pancreatic cancer risk with DPP-4 inhibitors [29]. Given their widespread use and overall safety profile, DPP-4 inhibitors remain a first-line or second-line treatment for many Japanese individuals with type 2 diabetes, though careful monitoring is warranted in specific high-risk populations. While meta-analyses of randomized controlled trials suggest a potentially increased risk of biliary diseases with DPP-4 inhibitors globally [13, 30], the absolute risk in Japanese populations appears unknown. However, clinicians should remain vigilant in high-risk individuals, particularly those with pre-existing gallbladder conditions. Further Japan-specific cohort studies are needed to clarify the clinical significance of this association.

Emerging evidence suggests that DPP-4 inhibitor use may be associated with an increased risk of bullous pemphigoid, an autoimmune blistering disorder characterized by edematous erythema and tense blisters [13, 31]. The risk is higher in males, older adults, HLA–DQB1*03:01 allele carriers [32], and those using less-selective DPP-4 inhibitors [33]. In suspected cases, dermatologic consultation and discontinuation of the DPP-4 inhibitor are advised. Cases of remitting seronegative symmetrical synovitis with pitting edema syndrome, presenting as symmetrical synovitis with dorsal hand and foot edema, have also been reported in individuals receiving DPP-4 inhibitors [13, 34]. Although a direct causal relationship remains unconfirmed, close monitoring is warranted.

## GLP-1 RAs

**Fig. 3:**
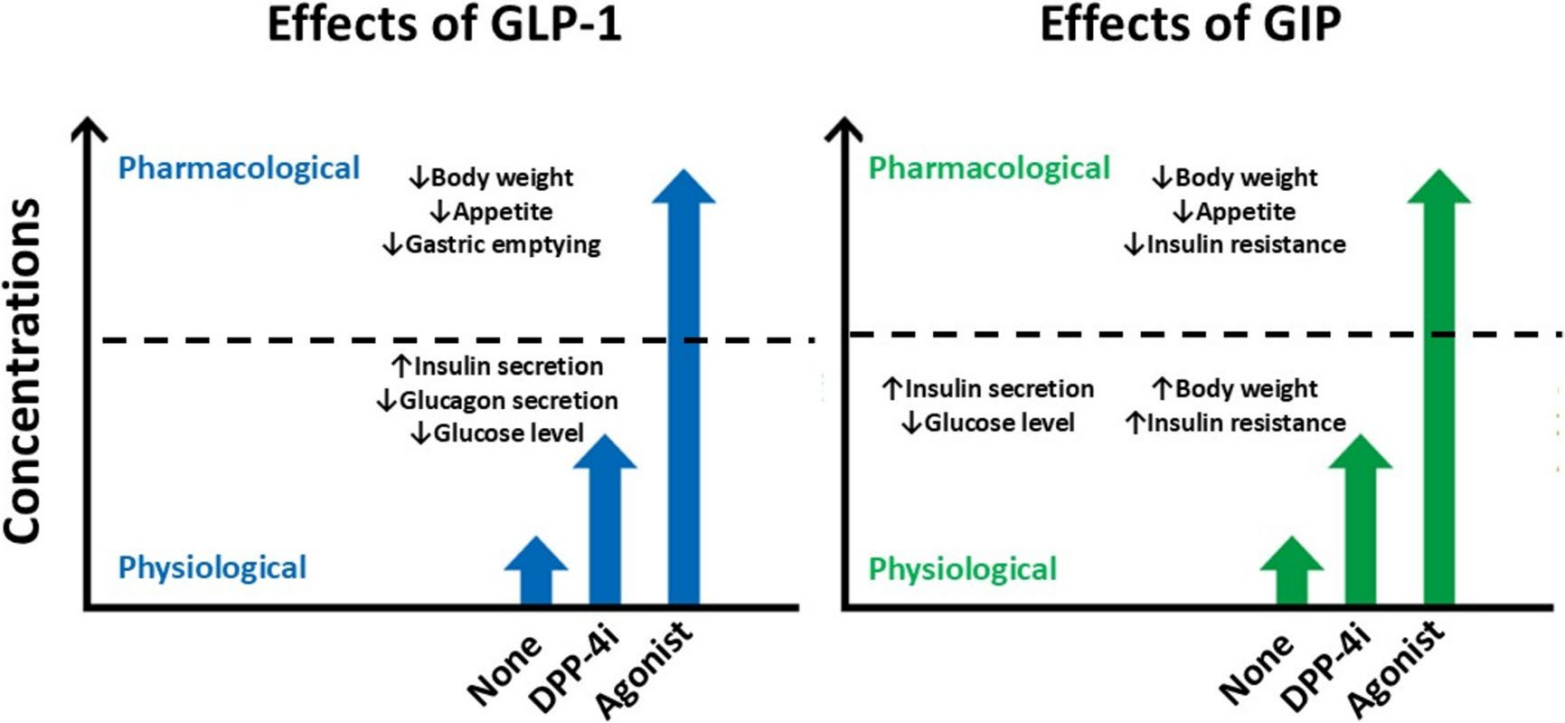
Different effects of glucagon-like peptide-1 (GLP-1) and glucose-dependent insulinotropic polypeptide (GIP) at physiological and pharmacological levels. Physiological levels of GLP-1, which can be maintained by DPP-4 inhibitors, enhance insulin secretion, suppress glucagon release, and improve glucose excursions. In contrast, pharmacological levels of GLP-1, achieved through GLP-1 receptor agonists or dual GIP/GLP-1 receptor agonists, additionally promote appetite suppression, body weight reduction, and delayed gastric emptying. Similarly, physiological levels of GIP, which can also be maintained by DPP-4 inhibitors, enhance insulin secretion and improve glucose excursions. However, pharmacological levels of GIP, achieved through dual GIP/GLP-1 receptor agonist, contribute to appetite suppression, body weight reduction, and increased gastric emptying. Notably, physiological levels of GIP may promote weight gain and subsequently exacerbate insulin resistance in individuals consuming diets rich in fats

Most of GLP-1 RAs available today are peptide-based agents resistant to DPP-4 degradation, with the majority formulated as injectables (Fig. 1). Like DPP-4 inhibitors, they ameliorate insulin secretion dysfunction and exert glucose-lowering effects (Fig. 2) [15], demonstrating particularly strong efficacy in East Asian populations, including Japanese individuals [4]. Beyond their glucose-lowering properties, GLP-1 RAs have gained significant attention as a therapeutic option for individuals with type 2 diabetes and obesity due to their appetite-suppressing effects (Fig. 3).

**Fig. 4:**
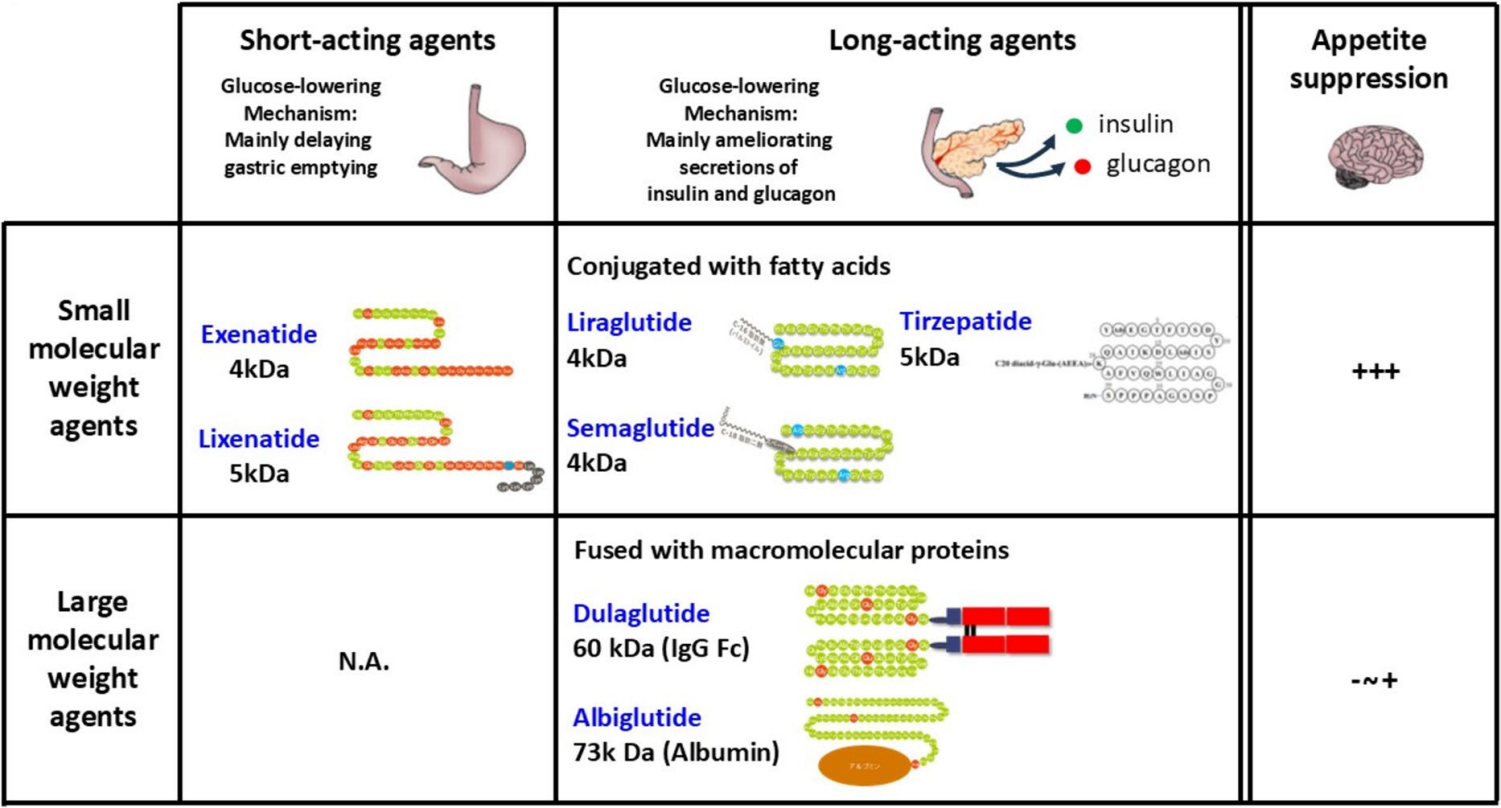
Differential effects of incretin receptor agonists based on duration of action and molecular weight. Short-acting incretin receptor agonists, such as exenatide and lixisenatide, primarily exert glucose-lowering effects by delaying gastric emptying. In contrast, long-acting incretin receptor agonists, including liraglutide, semaglutide, dulaglutide, and tirzepatide, improve glycaemia through the amelioration of insulin and glucagon secretion. Regarding molecular weight, small-molecule incretin receptor agonists (exenatide, lixisenatide, liraglutide, semaglutide, and tirzepatide) efficiently penetrate the brain, activating GLP-1 receptors to suppress appetite and promote weight loss. Conversely, large-molecule incretin receptor agonists (dulaglutide and albiglutide) have lower brain penetrability, resulting in a reduced impact on appetite suppression and body weight reduction

In addition, large-scale clinical trials have consistently reported their benefits in cardiovascular disease and diabetic kidney disease. Reflecting these advantages, the 2023 treatment algorithm for type 2 diabetes, published by the Japanese Diabetes Society, recommends GLP-1 RAs for patients with cardiovascular disease or chronic kidney disease, anticipating additional benefits [35, 36]. GLP-1 RAs are classified based on their structure and duration of action. Short-acting formulations (e.g., exenatide and lixisenatide) primarily delay gastric emptying, thereby improving postprandial glucose elevation [37, 38] (Fig. 4). Long-acting formulations (e.g., liraglutide, dulaglutide and semaglutide) predominantly enhance insulin secretion and suppress glucagon release, leading to sustained glycaemia [37, 38] (Fig. 4). A meta-analysis has demonstrated that long-acting GLP-1 RAs exert greater glucose-lowering and weight-reducing effects compared to short-acting formulations [39]. Moreover, once-weekly formulations have gained widespread adoption in recent years due to their convenience and improved adherence.

**Fig. 5:**
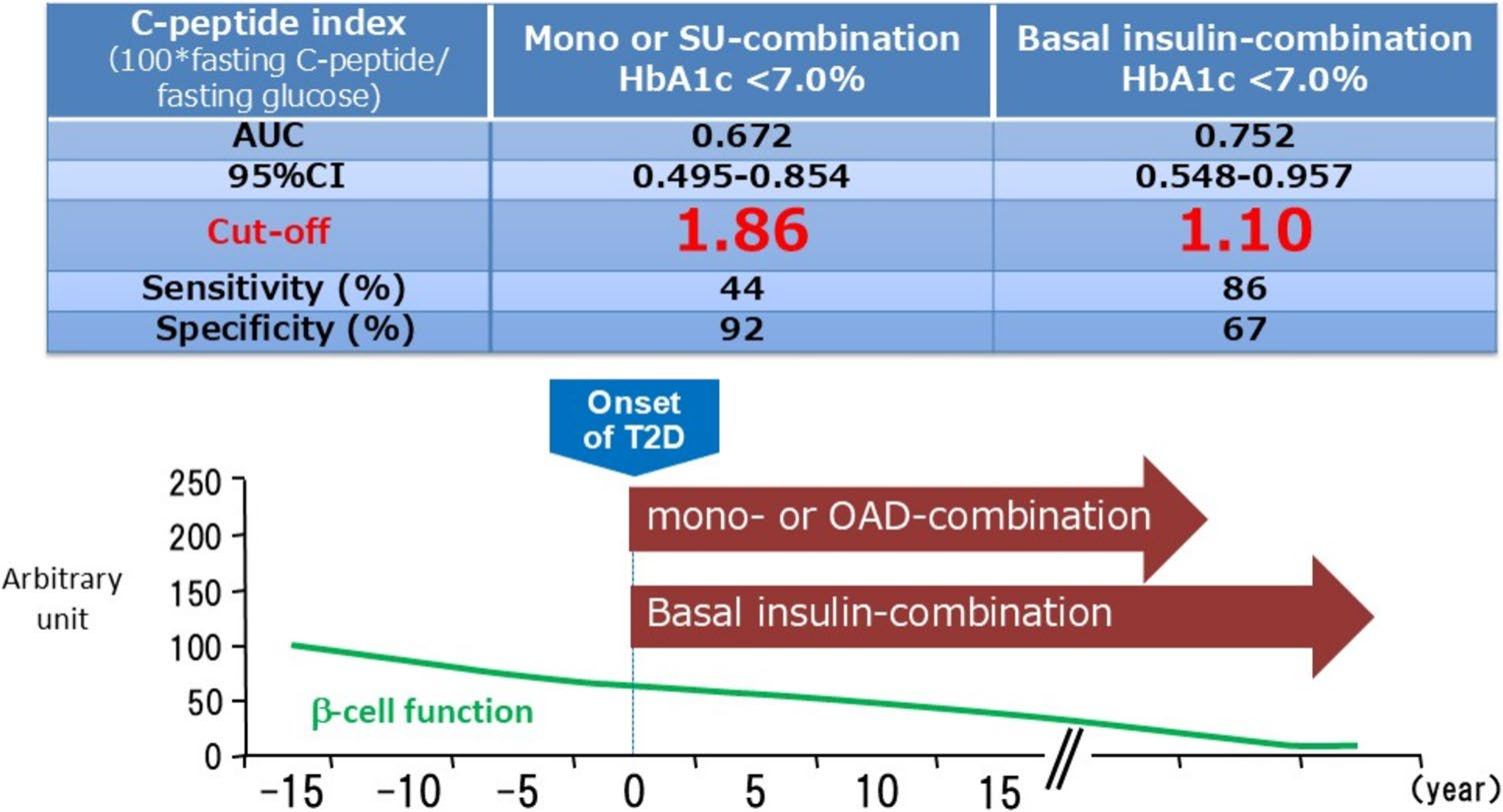
Glucose-lowering effects of glucagon-like peptide-1 receptor agonists (GLP-1 RAs) and remaining B-cell function. The glucose-lowering efficacy of long-acting GLP-1 RAs is strongly associated with residual pancreatic β-cell function and mass, a relationship extensively reported by multiple research groups. Given this dependency, early initiation is recommended to maximize therapeutic efficacy. It is suggested that the C-peptide index (CPI) is required to achieve an HbA1c level of <7.0% 1 year after GLP-1 RA initiation is lower when combined with insulin therapy. However, when CPI falls below a certain threshold, achieving glycemic targets becomes challenging, necessitating intensive insulin therapy. SU, sulfonylurea; T2D, type 2 diabetes; OAD, oral anti-diabetes drug

The glucose-lowering efficacy of long-acting GLP-1 RAs is strongly associated with residual pancreatic β-cell function and mass, a relationship extensively reported by multiple research groups [40–43]. This correlation was further validated in our preclinical study utilizing β-cell imaging [44], providing direct evidence of its significance. Given this dependency, early initiation is recommended to maximize therapeutic efficacy. Our analysis suggests that the C-peptide index (CPI) required to achieve an HbA1c level of <7.0% 1 year after GLP-1 RA initiation is lower when combined with insulin therapy (Fig. 5) [40, 41]. However, when CPI falls below a certain threshold, achieving glycemic targets becomes challenging, necessitating intensive insulin therapy [14, 41]. Notably, following the initial launch of the GLP-1 RA liraglutide in Japan, cases of diabetic ketoacidosis (DKA) and mortality were reported in insulin-dependent individuals who discontinued insulin therapy upon switching to liraglutide [13, 14]. As a result, in individuals with renal dysfunction, where accurate assessment of endogenous insulin secretion is challenging, a glucagon stimulation test is recommended. While glucagon stimulation testing is an effective method for evaluating residual pancreatic β-cell function, it may underestimate function in individuals receiving DPP-4 inhibitors [45], requiring cautious interpretation.

Given that the glucose-lowering effects of GLP-1 RAs are β-cell function-dependent, combination therapy with basal insulin is necessary in cases of significant β-cell dysfunction. Consequently, fixed-ratio combination therapies of insulin degludec/liraglutide and insulin glargine/lixisenatide have been developed as once-daily injection formulations (Fig. 1) [46–49]. Their effectiveness and safety have also been confirmed in real-world clinical settings [50–53]. In addition, a once-weekly insulin icodec/semaglutide combination is currently under global development, including in Japan [54]. Although fixed-ratio combination therapies maintain a constant GLP-1 RA-to-basal insulin ratio, individualized titration remains a challenge, potentially leading to excess or deficiency of either component. However, studies indicate that transitioning from intensive insulin therapy to combination therapy significantly improves treatment-related quality of life [55].

GLP-1 RAs promote weight reduction by acting on GLP-1 receptors in key brain regions involved in appetite regulation, including the arcuate nucleus of the hypothalamus and the nucleus tractus solitarius in the brainstem [56–58]. High-molecular-weight GLP-1 RAs, such as dulaglutide and albiglutide (not approved in Japan), exert their weight-reducing effects presumably due to limited brain penetration [59–61] (Fig. 4). In contrast, low-molecular-weight formulations are preferred in younger individuals with type 2 diabetes and obesity to maximize weight loss. Meanwhile, in older adults with type 2 diabetes, high-molecular-weight formulations are often selected to reduce the risk of frailty and sarcopenia. In addition, individuals who engage in stress-induced emotional eating may experience improved glycemic control but limited weight loss [62]. Our analysis supports these findings, suggesting that psychological interventions, such as counseling, may enhance treatment outcomes in such cases (manuscript in preparation).

GLP-1 RAs have been shown to provide cardiovascular and renal protection through multiple mechanisms, including suppression of chronic inflammation, improvement of lipid metabolism, reduction in blood pressure, and normalization of vascular endothelial function [5–7, 63]. Since 2008, the U.S. Food and Drug Administration has mandated cardiovascular outcome trials for new diabetes medications. A recent meta-analysis indicates that GLP-1 RAs significantly reduce major adverse cardiovascular events by 14%, cardiovascular death by 14%, hospitalization for heart failure by 13% [6]. In addition to their cardiovascular benefits, GLP-1 RAs also confer renal protection, demonstrating a 19% reduction in composite renal outcomes, a 16% reduction in end-stage kidney disease, a 21% reduction in kidney function decline [6]. Importantly, these benefits are independent of glucose-lowering effects, establishing them as "additional benefits" in diabetes management [35, 36]. Unfortunately, these additional benefits have not been consistently confirmed in Japanese individuals with type 2 diabetes, underscoring the need for further investigation.

Regarding adverse events associated with GLP-1 RAs, current evidence does not indicate an increased risk of pancreatic cancer or acute pancreatitis, unlike DPP-4 inhibitors. However, given that individuals with type 2 diabetes have a higher incidence of acute pancreatitis and pancreatic cancer, clinicians should remain vigilant when managing pancreatic health in this population [13]. Recent studies suggest a potential increase in the risk of biliary diseases with GLP-1 RAs [64, 65]. Therefore, caution is advised when prescribing these agents to individuals with pre-existing gallbladder conditions, despite the lack of Japan-specific evidence [13]. Further Japan-specific cohort studies are needed to clarify the clinical significance of this association.

Recent advancements have led to the development of oral GLP-1 RAs, broadening their clinical applications. Oral semaglutide, formulated with salcaprozate sodium to enhance mucosal absorption, has been developed (Fig. 1), and its effectiveness and safety have been evaluated in real-world clinical settings in Japan [59, 66–68]. It has been demonstrated that oral and injectable semaglutide achieve comparable efficacy and safety, provided that systemic drug concentrations remain equivalent [69]. However, when administered orally, semaglutide levels can vary among individuals, likely due to differences in absorption and adherence to dosing instructions. As a result, there has been a growing demand for oral GLP-1 RAs that offer greater ease of administration compared to oral semaglutide. In response, several promising low-molecular-weight, non-peptide GLP-1 receptor agonists are currently under development. Among them, orforglipron and danuglipron have demonstrated sustained GLP-1 receptor activation in global phase 2 trials, presenting potential new therapeutic options [70, 71].

## GIP/GLP-1 RAs

Currently, tirzepatide is the only GIP/GLP-1 RA developed and approved for clinical use in Japan (Fig. 1) [10, 72, 73]. It has a structure, where the N-terminal region resembles GIP, while the C-terminal region is similar to the GLP-1 RA exenatide [74]. The addition of a fatty acid moiety allows binding to albumin in the bloodstream, thereby extending its half-life (Fig. 4) [74]. This pharmacokinetic property enables once-weekly subcutaneous administration with sustained efficacy [74]. The *in vitro* study using cell lines expressing human GIP and GLP-1 receptors, tirzepatide exhibits comparable activity to GIP, whereas its activity on the GLP-1 receptor is approximately one-tenth that of GLP-1 [74]. However, tirzepatide possesses biased agonist properties toward the GLP-1 receptor [75], suggesting that it may sustainably activate the GLP-1 receptor *in vivo*. Given that GIP-mediated insulinotropic effects are diminished under chronic hyperglycemia [76, 77], the biased agonism of tirzepatide toward the GLP-1 receptor plays a crucial role in its efficacy. Once chronic hyperglycemia is corrected, GIP-mediated insulin secretion is restored [78], allowing tirzepatide to enhance insulin secretion through both the GIP and GLP-1 receptors (Fig. 2).

The SURPASS J-mono trial and SURPASS J-combo trial, conducted in Japan as part of the Phase 3 SURPASS program, demonstrated the efficacy and safety of tirzepatide in Japanese individuals with type 2 diabetes and obesity [72, 73]. After 1 year of tirzepatide treatment, more than half of the individuals achieved normoglycemia (HbA1c <5.7%). The JDS established a target HbA1c of <6.0% in the 2013 Kumamoto Declaration [79, 80], but achieving this without hypoglycemia had been challenging with conventional treatments. The introduction of tirzepatide may lead to a reassessment of the clinical significance of achieving normoglycemia for preventing and mitigating diabetes complications. The glucose-independent cardiovascular benefits of tirzepatide are currently being investigated in the SURPASS–CVOT trial, a major ongoing study evaluating its cardiovascular risk profile. This trial focuses on individuals with type 2 diabetes at high cardiovascular risk and assesses the impact of tirzepatide, compared to standard care, on major adverse cardiovascular events, including cardiovascular death, non-fatal myocardial infarction, and non-fatal stroke [81]. A post-hoc analysis of the SURPASS-4 trial demonstrated that tirzepatide attenuates the decline in both eGFRcreatinine and eGFRcystatin-C and significantly reduces the urinary albumin-to-creatinine ratio compared to insulin glargine [82, 83], suggesting a potential renoprotective effect in individuals with type 2 diabetes. Moreover, real-world evidence derived from the US Collaborative Network of TriNetX, which analyzed data from individuals with T2DM aged ≥18 years who initiated tirzepatide or a GLP-1 RA between June 1, 2022, and June 30, 2023, suggests that tirzepatide reduces the risk of all-cause mortality by 42%, MACE by 20%, and major adverse kidney events by 46% [84]. Further investigations are warranted to validate these findings in East Asian populations, including Japanese cohorts.

The most common adverse events associated with tirzepatide are gastrointestinal symptoms, including nausea and vomiting, which are similar to those observed with GLP-1 RAs [72, 73]. Although *in vitro* studies suggest that tirzepatide exhibits lower GLP-1 receptor activation compared to native GLP-1 [74], it is hypothesized to stimulate the GLP-1 receptor more strongly than semaglutide due to its biased agonism and higher dosing regimen (tirzepatide: 2.5–15 mg/week vs. semaglutide: 0.5–1.0 mg/week in Japan). Despite this, the incidence of gastrointestinal symptoms remains relatively low. One possible explanation is that tirzepatide activates GIP receptors on GABAergic neurons in the nucleus tractus solitarius and area postrema, which are involved in anti-emetic mechanisms, thereby reducing the occurrence of nausea and vomiting [85, 86].

A meta-analysis has reported a significantly higher incidence of gallstone formation and elevated serum lipase levels in the tirzepatide group compared to controls [87]. However, the incidence of cholecystitis and pancreatitis remains below 1%, with no significant difference between groups [87]. When tirzepatide is used without insulin, the incidence of hypoglycemia remains low [72, 73]. However, when combined with insulin or SUs, the incidence rises to approximately 10% [73, 88], underscoring the need for proper education on hypoglycemia prevention and management. In non-obese individuals, excessive weight loss may increase the risk of malnutrition and geriatric syndromes. Given these concerns, the JDS has issued the "Recommendations for Safe Use of Incretin-Based Therapies, Second Edition" (Table 1) [13] emphasizing that careful consideration of hypoglycemia risk, remaining β-cell function and mass, gastrointestinal side effects is essential when determining tirzepatide indications and treatment continuation.

## Emerging incretin-related therapeutics

Beyond conventional incretin-based therapies for type 2 diabetes and, more recently, obesity, the development of novel therapeutic agents is progressing rapidly. These innovations include small-molecule GLP-1 receptor agonists, as well as dual and triple receptor agonists targeting GIP, GLP-1, and glucagon. Notable examples include Survodutide, a GLP-1/glucagon receptor agonist, and Retatrutide, a GLP-1/GIP/glucagon receptor agonist [89, 90]. Alongside these incretin receptor agonists, GIP receptor antagonists have garnered significant interest, driven by evidence suggesting that endogenous GIP exacerbates obesity and insulin resistance in high-fat dietary conditions [19]. A GIP receptor-neutralizing antibody has demonstrated efficacy in suppressing diet-induced obesity in both murine models and non-human primates [91]. Furthermore, an engineered therapeutic, AMG-133, has been developed, consisting of a GIP receptor-neutralizing antibody covalently linked to two GLP-1 receptor agonist peptide molecules [92]. Currently, AMG-133 is undergoing a phase 2 clinical trial in adults with a BMI of ≥25 (overweight) or ≥30 (obese) and obesity-related metabolic disorders, including type 2 diabetes. Given its dual mechanism and promising preclinical results, AMG-133 is anticipated to be a significant advancement in obesity and metabolic disease management.

## Conclusion

Incretin-based therapies have revolutionized diabetes management both in Japan and globally. In Japan, where individuals aged 65 years and older account for over 70% of those with diabetes, a distinct non-obese phenotype with impaired insulin secretion is commonly observed. For these patients, DPP-4 inhibitors remain a cornerstone therapy, as they effectively enhance insulin secretion without increasing the risk of hypoglycemia. Conversely, among the growing population of younger adults with type 2 diabetes and obesity, GLP-1 RAs play a crucial role. These agents not only provide significant glycemic control and weight reduction but also offer renoprotective and cardioprotective benefits, particularly in the context of diabetic kidney disease and cardiovascular disorders. Looking ahead, emerging incretin-based agents, such as GIP/GLP-1 receptor co-agonists, hold promise for enhanced metabolic, weight, and cardiorenal outcomes. However, their long-term safety profiles and applicability, particularly in older adults and individuals with multiple comorbidities, require further investigation. The accumulation of long-term safety data and a comprehensive assessment of their clinical utility remain pressing challenges. To optimize therapeutic outcomes, a personalized medicine approach—tailoring treatment based on individual pathophysiology and lifestyle factors—is essential. In this context, the selection of incretin-related therapies should align with the guidelines outlined in the "Recommendations for the Safe Use of Incretin-Related Agents, Second Edition" by the committee on the safe use of medications, the Japan Diabetes Society (Table 1) [13].
